# Nonempirical Prediction of the Length-Dependent Ionization Potential in Molecular Chains

**DOI:** 10.1021/acs.jctc.4c00847

**Published:** 2024-08-13

**Authors:** Guy Ohad, Michal Hartstein, Tim Gould, Jeffrey B. Neaton, Leeor Kronik

**Affiliations:** † Department of Molecular Chemistry and Materials Science, Weizmann Institute of Science, Rehovoth 76100, Israel; ‡ Queensland Micro- and Nanotechnology Centre, Griffith University, Nathan QLD 4111, Australia; § Department of Physics, University of California, Berkeley, Berkeley, California 94720, United States; ∥ Materials Sciences Division, Lawrence Berkeley National Laboratory, Berkeley, California 94720, United States; ⊥ Kavli Energy NanoSciences Institute at Berkeley, University of California, Berkeley, Berkeley, California 94720, United States

## Abstract

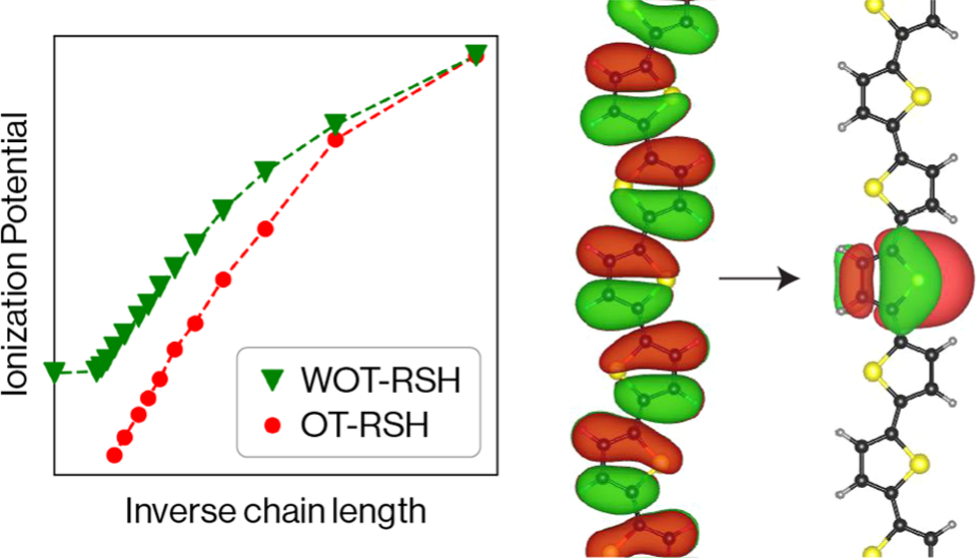

The ionization potential of molecular chains is well-known to be a tunable nanoscale property that exhibits clear quantum confinement effects. State-of-the-art methods can accurately predict the ionization potential in the small molecule limit and in the solid-state limit, but for intermediate, nanosized systems prediction of the evolution of the electronic structure between the two limits is more difficult. Recently, optimal tuning of range-separated hybrid functionals has emerged as a highly accurate method for predicting ionization potentials. This was first achieved for molecules using the ionization potential theorem (IPT) and more recently extended to solid-state systems, based on an *ansatz* that generalizes the IPT to the removal of charge from a localized Wannier function. Here, we study one-dimensional molecular chains of increasing size, from the monomer limit to the infinite polymer limit using this approach. By comparing our results with other localization-based methods and where available with experiment, we demonstrate that Wannier-localization-based optimal tuning is highly accurate in predicting ionization potentials for any chain length, including the nanoscale regime.

## I Introduction

Accurate prediction of the electronic properties of molecular and solid-state systems is of crucial importance in electronics and optoelectronics. In particular, the ionization potential (IP), electron affinity (EA), and therefore the fundamental gap, defined as the difference between the IP and EA, are key quantities in understanding materials and device properties. Ab initio many-body perturbation theory, typically within the *GW* approximation,^1−7^ can be a highly accurate method for predicting these quantities. But owing to its relatively high computational cost, there is an ongoing interest in developing accurate enough approximations within density functional theory (DFT) (see, e.g., refs (4,8−14)), that can serve as an inexpensive yet potentially still accurate alternative for computing these important properties.

Within the framework of DFT, we focus on optimal tuning (OT)^15^ of (screened)^16^ range-separated hybrid ((S)RSH) functionals, where screening is included for the case of bulk solids. OT-(S)RSH has been shown to be a highly accurate, nonempirical method for predicting electron removal/addition energies and therefore fundamental gaps for a variety of molecular systems (see, e.g., refs (15,17−24)) and molecular solids (see, e.g., refs (16,25−30)). Generally, RSH functionals allow for a different combination of exchange and correlation approximations at different ranges of electron–electron interactions and therefore offer flexibility in choosing appropriate functional parameters.^31−34^ OT-(S)RSH allows one to choose such parameters nonempirically by enforcing two conditions: the correct asymptotic behavior of long-range interactions^35−37^ and the ionization potential theorem (IPT).^35,36,38,39^ The latter has been shown to be particularly crucial not only for IP predictions, but also for accurate EA and therefore gap predictions.^40^

Unfortunately, optimal tuning based on straightforward application of the IPT fails in the solid-state limit. This is because, owing to the natural delocalization of electronic orbitals in this limit, the IPT is trivially satisfied for any choice of functional parameters, regardless of the accuracy (or lack thereof) of the obtained electronic structure.^41−44^ To overcome this significant limitation, a Wannier-localization-based optimal tuning of SRSH (WOT-SRSH) has been proposed.^45^ This approach enforces a generalized IPT *ansatz*,^46^ based on a constrained removal of charge from a localized Wannier function.^47^ WOT-SRSH has recently been shown to be highly successful in predicting band gaps and optical spectra of solids, both alone^45,48,49^ and as an optimal starting point to *GW* calculations,^49,50^ without any empiricism.

The success of OT-RSH in the molecular limit and WOT-SRSH in the solid-state limit immediately raises important questions as to the utilization of (W)OT approaches for intermediate-size systems. Specifically, for systems of increasing size, at which point does localization become necessary for optimal tuning? How do OT and WOT calculations compare with one another and how well do they predict the evolution of electronic properties with system size, compared to experiment and/or other benchmark calculations?

A class of systems where such questions arise naturally and can be examined systematically is that of linear oligomers, i.e., linear molecular chains composed of a variable number of repeating units of a given monomer. Indeed, such systems have been previously used to test the accuracy of various approaches within DFT.^13,17,51−57^

Here, we use these benchmark systems to answer the above questions by employing OT- and WOT-RSH to compute the IPs of three different one-dimensional molecular chains of increasing length, from the monomer limit to the infinite polymer limit. By comparing our results to other methods and to experiment where available, we show that OT- and WOT-RSH yield essentially identical results for shorter chains, but deviate from each other for larger chains, with WOT-RSH yielding a correct convergence to the infinite polymer limit and providing consistently more accurate results than OT-RSH, as compared to reference theoretical results.

## II Methods

### II.I Benchmark Systems

**Figure 1:**
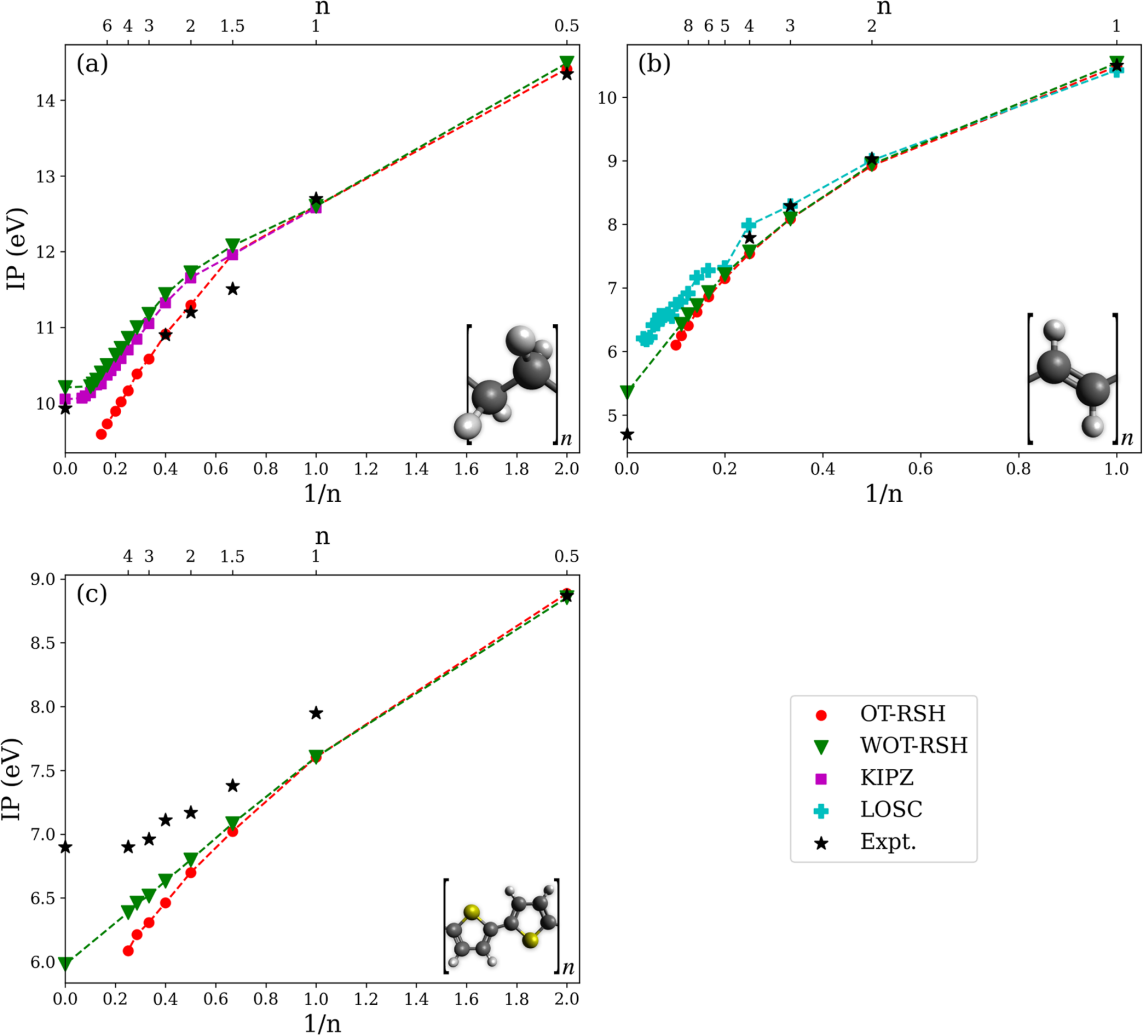
Ionization potential as a function of *n*, the number of repeating units (top axis), and the inverse of *n* (bottom axis), for (a) alkanes, (b) *trans*-oligoacetylenes (tOAs), and (c) oligothiophenes (OLTs). Computed results are given by the negative of the HOMO energy based on OT-RSH (red circles), WOT-RSH (green triangles), KIPZ (magenta squares, from ref (59)), and LOSC (cyan plus signs, from ref (13)). Experimental results (black stars) are taken from the following sources: For alkanes: *n* = 0.5: ref (73), *n* = 1: ref (74), *n* = 1.5, 2, 2.5: ref (75), *n* → ∞: refs (76−78). For tOAs: *n* = 1,2: ref (75), *n* = 3: ref (79), *n* = 4: ref (80), *n* → ∞: ref (81). For OLTs: refs (51,82). Inset: schematic view of the repeating unit of each chain, showing carbon atoms in black, hydrogen atoms in gray, and sulfur atoms in yellow.

We study three types of one-dimensional molecular chains: Linear alkanes, *trans*-oligoacetylenes (tOAs), and oligothiophenes (OLTs), the chemical formulas of which are C_2*n*_H_4*n*+2_, C_2*n*_H_2*n*+2_, and C_8*n*_H_4*n*+2_S_2*n*_, respectively (see inset of Figure 1). Half-integer *n* values correspond to an odd number of carbon atoms for the alkanes and tOAs and an odd number of sulfur atoms for the OLTs. For clarity, throughout we use the term “polymer” to refer only to the limit of *n* → ∞, namely polyethylene, *trans*-polyacetylene and polythiophene, respectively. Molecular geometry was optimized using molecular mechanics, without further optimization at the DFT level (to which spectral properties are sensitive^58^), so that the effect of orbital delocalization is due to chain length increase alone. This follows a similar practice in refs (55,59). See the Supporting Information (SI)(60) for more details.

### II.II Range-Separated Hybrid Functionals

In RSH functionals,^61,62^ the Coulomb operator is partitioned into two terms, typically by exploiting the error function, erf, in the form
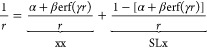
1where *r* is the interelectronic distance and α, β, γ are free parameters. While eq 1 represents a trivial identity, this split of the Coulomb repulsion allows for use of different approximations for the electron exchange associated with each term. For the first term, we use Fock (exact) exchange (xx), whereas for the second we use semilocal exchange (SLx). This leads to two limiting-case fractions of exact exchange: α for short-range (SR) interactions (*r* → 0) and α + β for long-range (LR) interactions (*r* → ∞). These two limits are interpolated smoothly via the error function, with the transition governed by the range-separation parameter, γ. Accordingly, the exchange energy of RSH is expressed as
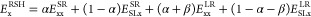
2

In this work, we choose α as 0.25 throughout, as in global and short-range hybrid functionals,^63−65^ in order to retain a useful balance between exchange and correlation in the short-range.^66^ The correct asymptotic limit of the potential is attained by setting α + β = 1/ε, where ε is the scalar dielectric constant.^25^ In this work we set ε = 1, the appropriate choice for an isolated molecule in vacuum,^35−37^ which is correct also for the polymers, where asymptotic screening vanishes owing to the low dimensionality (see, e.g., refs (67−69), for two dimensions). We use the range-separated version^70,71^ of the Perdew–Burke–Ernzerhof (PBE) exchange functional^72^ to treat the semilocal exchange components in the RSH, along with full PBE semilocal correlation. Nonempirical methods employed to select γ are discussed below.

Once the three parameters are selected, one can compute any property of interest. In this work, we focus on the eigenvalue corresponding to the highest occupied molecular orbital (HOMO), the negative of which provides a prediction for the IP in an optimally tuned functional.

### II.III Optimal Tuning of RSH

As mentioned in the introduction, in the OT-RSH approach γ is selected to enforce the IPT, which is an exact physical condition in DFT. Here, we enforce the IPT for the neutral system, namely we seek γ such that
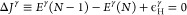
3where *E*^γ^(*N*) and *E*^γ^(*N* – 1) are the total ground-state energies for the neutral system with *N* electrons and the singly ionized cation, respectively, and ϵ_H_^γ^ is the HOMO eigenvalue for the *N*-electron system. See the SI(60) for more computational details.

### II.IV Wannier-Localization-Based Optimal Tuning of RSH

As mentioned above, in the bulk limit the IPT is trivially satisfied for any choice of γ, which precludes the predictive selection of a unique range-separation value based on eq 3. Many authors have explored localized orbitals as a means of circumventing this limitation of the IPT, in the context of different approaches within DFT.^11,13,45,46,56,59,83−95^ In the context of SRSH functionals, Wing et al.^45^ adopted an *ansatz*(46) that generalizes the IPT to the removal of an electron from a state that corresponds to a localized Wannier function, namely
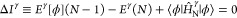
4where ϕ is the maximally localized Wannier function^47^ for which the expectation energy with respect to the DFT Hamiltonian of the *N*-electron system, ⟨ϕ|*Ĥ*_N_^γ^|ϕ⟩, is the largest. *E*^γ^[ϕ](*N* – 1) is the total energy of the constrained (*N* – 1)-electron system, which differs from the ground-state (*N* – 1)-electron system in that an electron has been removed from the Wannier function.^45^ This constraint is imposed via a Lagrange multiplier that controls the occupancy of the Wannier function, which is constructed of the occupied-orbital manifold using the PBE functional. We emphasize that for finite systems, maximally localized Wannier functions reduce to Foster-Boys orbitals,^96−99^ and can be viewed as their solid-state equivalent.^47^ We use the term Wannier functions throughout, because it applies in both the molecular and solid-state limits and because the localized orbitals that we use are generated with the wannier90 code.^100^ Additional computational details are given in the SI.^60^

## III Results and Discussion

Figure 1 shows the computed IP, based on the negative of both the OT-RSH and WOT-RSH HOMO energy, as a function of the inverse of *n*, the number of repeating units, for each of the three systems studied in this work. These results are compared with those obtained from two other localization-based methods: the integer Koopmans plus Perdew–Zunger (KIPZ) correction method for the alkanes, taken from ref (59), and the localized orbital scaling correction (LOSC) approach for the tOAs, taken from ref (13). The computational results are further compared with experiment where available.

**Figure 2:**
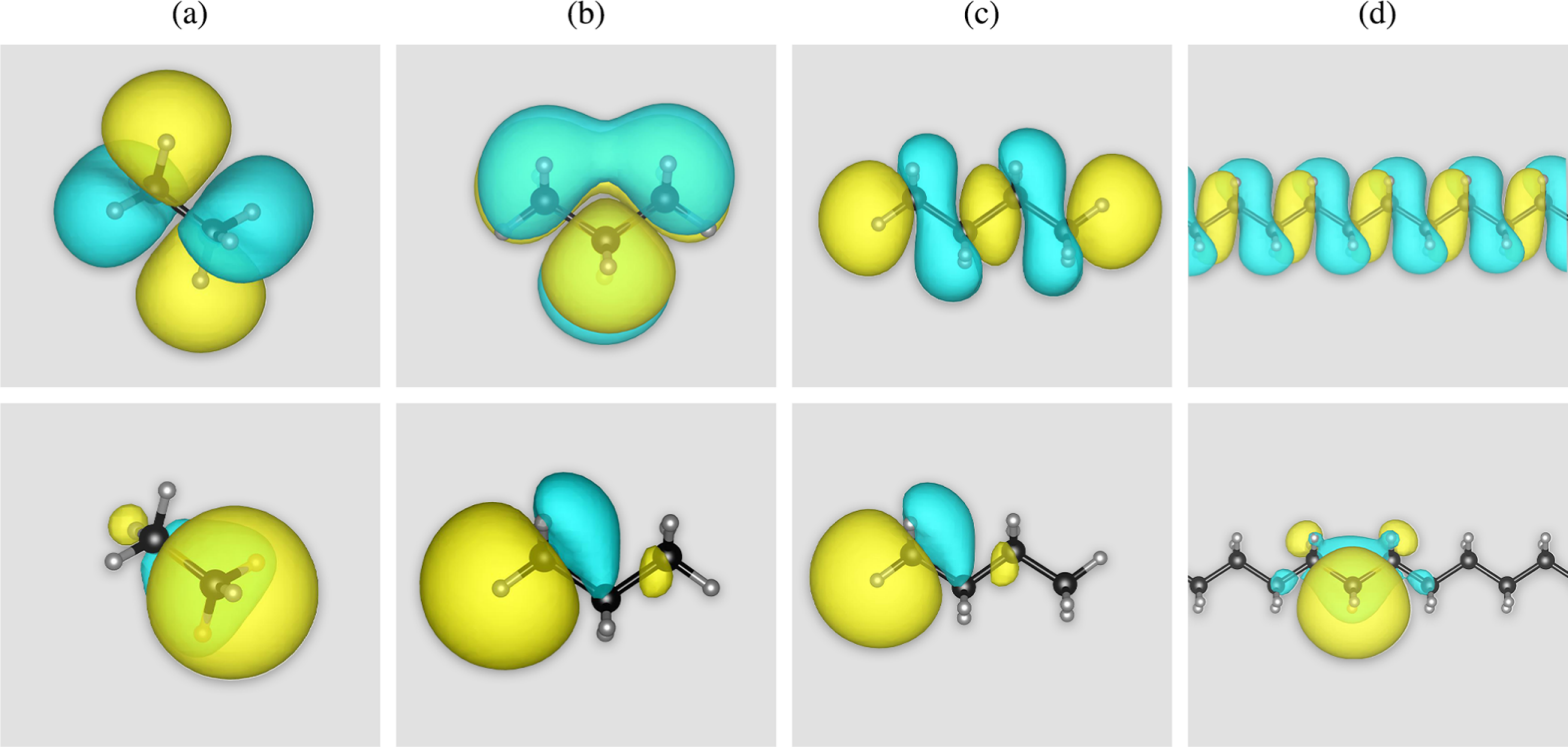
HOMO (top row) and highest expectation energy Wannier function (bottom row) for selected alkanes: (a) ethane (*n* = 1), (b) propane (*n* = 1.5), (c) butane (*n* = 2), and (d) polyethylene (*n* → ∞). Carbon and hydrogen atoms are shown in black and gray, respectively. The wave function isosurface is shown in light blue and yellow for a value of 2.0.

First, we compare the OT-RSH and WOT-RSH results. We observe two trends that are common to all three systems. First, the two methods predict essentially identical IPs for the shorter chains, where the HOMOs are “naturally” localized owing to the small system size. This is a significant observation, because it demonstrates the validity and generality of the IPT *ansatz* used in WOT-RSH, even in a realm for which it was not designed and in which it is not strictly necessary. Second, the deviation between the two methods increases with chain length and becomes as large as ∼0.8 eV for the case of alkanes with *n* = 7. We attribute this to the delocalization of the HOMO in the longer chains, shown in Figure 2 for selected alkanes and in the SI for selected tOAs and OLTs. This delocalization ultimately prevents the use of OT-RSH altogether for large enough chains, as the IPT is approaching the point where it is trivially obeyed and a numerically stable determination of the range-separation parameter is no longer possible. In contrast, the WOT-RSH relies on a Wannier function that is localized by construction and changes little with *n*, as also shown in Figure 2 for selected alkanes and in the SI for selected tOAs and OLTs. As a result, the WOT-RSH procedure is numerically stable and physically meaningful for any *n*, including *n* → ∞, i.e., the polymer limit.

Interestingly, while the trend of OT- and WOT-RSH results deviating from one another is common to the three systems, it appears to be more abrupt for the alkanes, where the deviation starts already at relatively short chains, but more gradual and at larger *n* for the two other systems. We note that the abrupt deviation in the alkanes occurs between *n* = 1.5 and *n* = 2. This can be associated with an abrupt change in the symmetry of the HOMO, owing to orbital reordering, between these two chains, as demonstrated in Figure 2(b,c). The same symmetry as in *n* = 2 is then maintained for all *n* > 2. The symmetry of the HOMO for the tOAs and OLTs, on the other hand, is unchanged for all *n*, as demonstrated in the SI.

Next, we compare our results to benchmark computational data. As shown in Figure 1, the general trend of the IP saturating with increasing chain length, up to the polymer limit, is clearly captured. This trend is less observed in the tOAs, in agreement with the results of ref (55), which showed that the saturation occurs in longer chain lengths that are outside the range studied in this work. Furthermore, the WOT-RSH results agree very well quantitatively with previous localization-based schemes—to ∼0.1 eV with KIPZ results and ∼0.2 eV with LOSC, on average. Conversely, and as expected based on the above discussion, the OT-RSH results do not extrapolate to the correct polymer limit.

Finally, we compare the computed results to experimental ones. For alkanes, the experimental results agree well with all theoretical methods for *n* = 0.5 and *n* = 1. For *n* = 1.5, all theoretical methods appear to agree, but predict a value larger than experiment by ∼0.5 eV. For *n* = 2 and *n* = 2.5, WOT-RSH and KIPZ overestimate experiment by a similar amount, while OT-RSH is in better agreement with it. This, however, may be accidental, given the fact that the *n* = 1.5 result of OT-RSH overestimates experiment. The IP for *n* → ∞, polyethylene, agrees well with several experimental estimations.^76−78^

For the tOAs, agreement with existing experimental values for finite chains is consistently good for all theoretical methods. The experimental value for *trans*-polyacetylene is taken from solid-state measurements, where the IP can be smaller by hundreds of meV from the gas-phase IP,^81^ possibly explaining the deviation from the WOT-RSH prediction. For the OLTs, with the exception of the *n* = 0.5 monomer, our results underestimate experimental ones by more than 0.3 eV. Whether this discrepancy is related to structural differences, thermal effects, experimental uncertainties, or theoretical limitations is at present unknown. Even so, the WOT-RSH results agree qualitatively and semiquantitatively with the experimental ones, whereas the OT-RSH results do not. Overall, then, the WOT-RSH results provide good agreement with experimental trends, where available, throughout.

**Figure 3:**
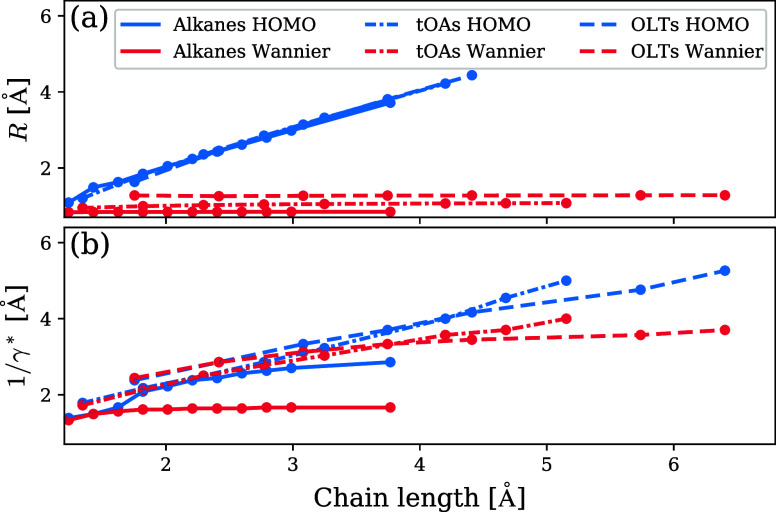
(a) Spatial spread, *R*, and (b) optimal tuning length, 1/γ*, as a function of effective chain length (see text for details), for the three systems studied in this work. Blue curves correspond to highest-occupied molecular orbitals and the OT approach, red curves correspond to Wannier functions and the WOT approach.

In order to obtain a deeper understanding of the similarities and differences between OT-RSH and WOT-RSH, we further analyze quantities that are central to the optimal tuning procedure. Figure 3(a) shows the spatial spread, *R*, which is the square root of the second moment of the position operator,^47^ and therefore a measure of the degree of localization (see the SI for additional details). It is shown for all systems studied in this work, as a function of effective chain length, for both the HOMO used in the OT approach (eq 3) and for the Wannier function used in the WOT approach (eq 4). Figure 3(b) similarly shows the optimal tuning length, namely the inverse of the optimally tuned range-separation parameter γ*. Here, the effective chain length is defined via *c*_0_ + *nc*_1_, where *c*_0_ and *c*_1_ have been determined through a linear fit of *R* of the HOMO to *n*. This means that the effective chain length is simply the linear fit of *R*, based on the (justified) assumption that the HOMO is delocalized across the entire chain.

The first observation of note is that the HOMOs and Wannier functions exhibit different spreads already for short chains. Nonetheless, for the same shorter chains γ* values are nearly identical in OT and WOT. We further tested this result by applying the same methodology to benzene. This yielded spreads of 1.7 and 1.1 Å for the HOMO and Wannier function, respectively, but similar γ* values of 0.42 and 0.39 Å^–1^ from OT and WOT, respectively. This indicates that the energy differences caused by the different densities used in the OT and WOT procedures are compensated for, consistently, by the different tuning criteria of the two approaches. Thus, using WOT over OT for small molecules is not strictly necessary but is useful for consistency along the evolution of system size.

The second observation of note is that the spread of the HOMO continues to increase as chain length increases, reflecting the increased delocalization observed in Figure 2, with a concomitant increase in the optimal tuning length. In fact, there is an almost perfectly linear relation between orbital spread and optimal tuning length for the longer chains in all three molecule types. This validates, by providing a more quantitative framework, the conceptual argument regarding the failure of OT for increasingly larger systems due to a uniform removal of an electron from the entire system.

A third observation of note is that despite the spread of the Wannier functions attaining its *n* → ∞ limit already for very short chains (manifested as near constant red plots in Figure 3(a)), the optimal tuning length continues to vary more significantly as the chain length increases. This can be rationalized by considering that the WOT procedure represents the removal of a localized electron, but that electron is still effectively removed from the entire molecule. This shows that the WOT procedure is indeed capable of capturing features of the “true” potential, including those on a larger scale than the orbital itself. Taken together, the above observations explain the utility of the WOT approach throughout the evolution of the chain length.

## IV Conclusions

We have compared two optimal tuning methods of RSH, namely OT-RSH and WOT-RSH, for the computation of the IP of one-dimensional molecular chains of increasing length. We have demonstrated the known failure of OT-RSH for long chains, owing to orbital delocalization. We found, however, that WOT-RSH is successful in predicting accurate IPs throughout the evolution of the chain length, not only in the polymer limit for which it was originally designed, but throughout the entire range of oligomers, from monomer to polymer. Specifically, WOT-RSH results agree with both experimental trends and past localization-based computational schemes. This provides a first step in the application of optimal tuning to nanosized objects where neither the molecular nor the bulk limits apply.

## Supporting Information Available

Computational details; comparison between homo and Wannier function (PDF)

## References

[ref1] Hedin L. New method for calculating the one-particle Green’s function with application to the electron-gas problem. Phys. Rev. 1965, 139 (3A), A796 10.1103/PhysRev.139.A796.

[ref2] Hybertsen M. S.; Louie S. G. First-Principles theory of quasiparticles: Calculation of band gaps in semiconductors and insulators. Phys. Rev. Lett. 1985, 55 (13), 1418–1421. 10.1103/PhysRevLett.55.1418. 10031814

[ref3] Aulbur W. G.; Jönsson L.; Wilkins J. W. Quasiparticle Calculations in Solids. In Solid State Physics; Ehrenreich H.; Spaepen F., Eds.; Academic Press, 2000; Vol. 54, pp 1–218.

[ref4] Onida G.; Reining L.; Rubio A. Electronic excitations: density-functional versus many-body Green’s-function approaches. Rev. Mod. Phys. 2002, 74 (2), 601 10.1103/RevModPhys.74.601.

[ref5] Louie S. G.; Rubio A. Quasiparticle and Optical Properties of Solids and Nanostructures: The GW-BSE Approach. In Handbook of Materials Modeling; Springer, 2005; pp 215–240.

[ref6] Golze D.; Dvorak M.; Rinke P. The GW compendium: A practical guide to theoretical photoemission spectroscopy. Front. Chem. 2019, 7, 377 10.3389/fchem.2019.00377. 31355177 PMC6633269

[ref7] Martin R. M.; Reining L.; Ceperley D. M. Interacting Electrons: Theory and Computational Approaches; Cambridge University Press: Cambridge, 2016.

[ref8] Kümmel S.; Kronik L. Orbital-dependent density functionals: Theory and applications. Rev. Mod. Phys. 2008, 80, 3–60. 10.1103/RevModPhys.80.3.

[ref9] Tran F.; Blaha P. Accurate band gaps of semiconductors and insulators with a semilocal exchange-correlation potential. Phys. Rev. Lett. 2009, 102, 226401 10.1103/PhysRevLett.102.226401. 19658882

[ref10] Perdew J. P.; Yang W.; Burke K.; Yang Z.; Gross E. K. U.; Scheffler M.; Scuseria G. E.; Henderson T. M.; Zhang I. Y.; Ruzsinszky A.; Peng H.; Sun J.; Trushin E.; Görling A. Understanding band gaps of solids in generalized Kohn–Sham theory. Proc. Natl. Acad. Sci. U.S.A. 2017, 114 (11), 2801–2806. 10.1073/pnas.1621352114. 28265085 PMC5358356

[ref11] Miceli G.; Chen W.; Reshetnyak I.; Pasquarello A. Nonempirical hybrid functionals for band gaps and polaronic distortions in solids. Phys. Rev. B 2018, 97, 121112 10.1103/PhysRevB.97.121112.

[ref12] Colonna N.; Nguyen N. L.; Ferretti A.; Marzari N. Koopmans-compliant functionals and potentials and their application to the GW100 test set. J. Chem. Theory Comput. 2019, 15 (3), 1905–1914. 10.1021/acs.jctc.8b00976. 30640457

[ref13] Li C.; Zheng X.; Su N. Q.; Yang W. Localized orbital scaling correction for systematic elimination of delocalization error in density functional approximations. Natl. Sci. Rev. 2018, 5 (2), 203–215. 10.1093/nsr/nwx111.

[ref14] Lebeda T.; Aschebrock T.; Sun J.; Leppert L.; Kümmel S. Right band gaps for the right reason at low computational cost with a meta-GGA. Phys. Rev. Mater. 2023, 7 (9), 093803 10.1103/PhysRevMaterials.7.093803.

[ref15] Kronik L.; Stein T.; Refaely-Abramson S.; Baer R. Excitation gaps of finite-sized systems from optimally tuned range-separated hybrid functionals. J. Chem. Theory Comput. 2012, 8 (5), 1515–1531. 10.1021/ct2009363. 26593646

[ref16] Kronik L.; Kümmel S. Dielectric screening meets optimally tuned density functionals. Adv. Mater. 2018, 30 (41), 1706560 10.1002/adma.201706560. 29665112

[ref17] Stein T.; Eisenberg H.; Kronik L.; Baer R. Fundamental gaps in finite systems from eigenvalues of a generalized Kohn-Sham method. Phys. Rev. Lett. 2010, 105, 266802 10.1103/PhysRevLett.105.266802. 21231698

[ref18] Refaely-Abramson S.; Baer R.; Kronik L. Fundamental and excitation gaps in molecules of relevance for organic photovoltaics from an optimally tuned range-separated hybrid functional. Phys. Rev. B 2011, 84, 075144 10.1103/PhysRevB.84.075144.

[ref19] Autschbach J.; Srebro M. Delocalization error and “functional tuning” in Kohn–Sham calculations of molecular properties. Acc. Chem. Res. 2014, 47 (8), 2592–2602. 10.1021/ar500171t. 24968277

[ref20] Phillips H.; Zheng Z.; Geva E.; Dunietz B. D. Orbital gap predictions for rational design of organic photovoltaic materials. Org. Electron. 2014, 15 (7), 1509–1520. 10.1016/j.orgel.2014.03.040.

[ref21] Foster M. E.; Azoulay J. D.; Wong B. M.; Allendorf M. D. Novel metal–organic framework linkers for light harvesting applications. Chem. Sci. 2014, 5, 2081–2090. 10.1039/C4SC00333K.

[ref22] Körzdörfer T.; Brédas J.-L. Organic electronic materials: Recent advances in the DFT description of the ground and excited states using tuned range-separated hybrid functionals. Acc. Chem. Res. 2014, 47 (11), 3284–3291. 10.1021/ar500021t. 24784485

[ref23] Faber C.; Boulanger P.; Attaccalite C.; Duchemin I.; Blase X. Excited states properties of organic molecules: From density functional theory to the GW and Bethe-Salpeter Green’s function formalisms. Philos. Trans. R. Soc., A 2014, 372 (2011), 20130271 10.1098/rsta.2013.0271. 24516185

[ref24] Alipour M.; Mohseni S. Shedding light on the accuracy of optimally tuned range-separated approximations for evaluating oxidation potentials. J. Phys. Chem. A 2017, 121 (21), 4189–4201. 10.1021/acs.jpca.7b03811. 28513157

[ref25] Refaely-Abramson S.; Sharifzadeh S.; Jain M.; Baer R.; Neaton J. B.; Kronik L. Gap renormalization of molecular crystals from density-functional theory. Phys. Rev. B 2013, 88, 081204 10.1103/PhysRevB.88.081204.

[ref26] Lüftner D.; Refaely-Abramson S.; Pachler M.; Resel R.; Ramsey M. G.; Kronik L.; Puschnig P. Experimental and theoretical electronic structure of quinacridone. Phys. Rev. B 2014, 90, 075204 10.1103/PhysRevB.90.075204.

[ref27] Kronik L.; Neaton J. B. Excited-state properties of molecular solids from first principles. Annu. Rev. Phys. Chem. 2016, 67, 587–616. 10.1146/annurev-physchem-040214-121351. 27090844

[ref28] Bhandari S.; Cheung M. S.; Geva E.; Kronik L.; Dunietz B. D. Fundamental gaps of condensed-phase organic semiconductors from single-molecule calculations using polarization-consistent optimally tuned screened range-separated hybrid functionals. J. Chem. Theory Comput. 2018, 14 (12), 6287–6294. 10.1021/acs.jctc.8b00876. 30444365

[ref29] Coropceanu V.; Chen X.-K.; Wang T.; Zheng Z.; Brédas J.-L. Charge-transfer electronic states in organic solar cells. Nat. Rev. Mater. 2019, 4 (11), 689–707. 10.1038/s41578-019-0137-9.

[ref30] Franco L. R.; Marchiori C.; Araujo C. M. Unveiling the impact of exchange-correlation functionals on the description of key electronic properties of non-fullerene acceptors in organic photovoltaics. J. Chem. Phys. 2023, 159, 204110 10.1063/5.0163180. 38018752

[ref31] Toulouse J.; Colonna F.; Savin A. Long-range–short-range separation of the electron-electron interaction in density-functional theory. Phys. Rev. A 2004, 70 (6), 062505 10.1103/PhysRevA.70.062505.

[ref32] Vydrov O. A.; Heyd J.; Krukau A. V.; Scuseria G. E. Importance of short-range versus long-range Hartree-Fock exchange for the performance of hybrid density functionals. J. Chem. Phys. 2006, 125, 074106 10.1063/1.2244560. 16942321

[ref33] Chai J.-D.; Head-Gordon M. Systematic optimization of long-range corrected hybrid density functionals. J. Chem. Phys. 2008, 128, 084106 10.1063/1.2834918. 18315032

[ref34] Rohrdanz M. A.; Martins K. M.; Herbert J. M. A long-range-corrected density functional that performs well for both ground-state properties and time-dependent density functional theory excitation energies, including charge-transfer excited states. J. Chem. Phys. 2009, 130, 054112 10.1063/1.3073302. 19206963

[ref35] Levy M.; Perdew J. P.; Sahni V. Exact differential equation for the density and ionization energy of a many-particle system. Phys. Rev. A 1984, 30, 2745–2748. 10.1103/PhysRevA.30.2745.

[ref36] Almbladh C.-O.; von Barth U. Exact results for the charge and spin densities, exchange-correlation potentials, and density-functional eigenvalues. Phys. Rev. B 1985, 31, 3231–3244. 10.1103/PhysRevB.31.3231. 9936207

[ref37] Kronik L.; Kümmel S. Piecewise linearity, freedom from self-interaction, and a Coulomb asymptotic potential: three related yet inequivalent properties of the exact density functional. Phys. Chem. Chem. Phys. 2020, 22 (29), 16467–16481. 10.1039/D0CP02564J. 32661542

[ref38] Perdew J. P.; Parr R. G.; Levy M.; Balduz J. L. Density-functional theory for fractional particle number: Derivative discontinuities of the energy. Phys. Rev. Lett. 1982, 49, 1691–1694. 10.1103/PhysRevLett.49.1691.

[ref39] Perdew J. P.; Levy M. Comment on “Significance of the highest occupied Kohn-Sham eigenvalue. Phys. Rev. B 1997, 56, 16021–16028. 10.1103/PhysRevB.56.16021.

[ref40] Stein T.; Autschbach J.; Govind N.; Kronik L.; Baer R. Curvature and frontier orbital energies in density functional theory. J. Phys. Chem. Lett. 2012, 3 (24), 3740–3744. 10.1021/jz3015937. 26291104

[ref41] Mori-Sánchez P.; Cohen A. J.; Yang W. Localization and delocalization errors in density functional theory and implications for band-gap prediction. Phys. Rev. Lett. 2008, 100, 146401 10.1103/PhysRevLett.100.146401. 18518055

[ref42] Kraisler E.; Kronik L. Fundamental gaps with approximate density functionals: The derivative discontinuity revealed from ensemble considerations. J. Chem. Phys. 2014, 140 (18), 18A540 10.1063/1.4871462. 24832348

[ref43] Vlček V.; Eisenberg H. R.; Steinle-Neumann G.; Kronik L.; Baer R. Deviations from piecewise linearity in the solid-state limit with approximate density functionals. J. Chem. Phys. 2015, 142, 034107 10.1063/1.4905236. 25612689

[ref44] Görling A. Exchange-correlation potentials with proper discontinuities for physically meaningful Kohn-Sham eigenvalues and band structures. Phys. Rev. B 2015, 91, 245120 10.1103/PhysRevB.91.245120.

[ref45] Wing D.; Ohad G.; Haber J. B.; Filip M. R.; Gant S. E.; Neaton J. B.; Kronik L. Band gaps of crystalline solids from Wannier-localization-based optimal tuning of a screened range-separated hybrid functional. Proc. Natl. Acad. Sci. U.S.A. 2021, 118, e2104556118 10.1073/pnas.2104556118. 34417292 PMC8403912

[ref46] Ma J.; Wang L.-W. Using Wannier functions to improve solid band gap predictions in density functional theory. Sci. Rep. 2016, 6 (1), 24924 10.1038/srep24924. 27114185 PMC4845067

[ref47] Marzari N.; Mostofi A. A.; Yates J. R.; Souza I.; Vanderbilt D. Maximally localized Wannier functions: Theory and applications. Rev. Mod. Phys. 2012, 84, 1419–1475. 10.1103/RevModPhys.84.1419.

[ref48] Ohad G.; Wing D.; Gant S. E.; Cohen A. V.; Haber J. B.; Sagredo F.; Filip M. R.; Neaton J. B.; Kronik L. Band gaps of halide perovskites from a Wannier-localized optimally tuned screened range-separated hybrid functional. Phys. Rev. Mater. 2022, 6 (10), 104606 10.1103/PhysRevMaterials.6.104606.

[ref49] Ohad G.; Gant S. E.; Wing D.; Haber J. B.; Camarasa-Gómez M.; Sagredo F.; Filip M. R.; Neaton J. B.; Kronik L. Optical absorption spectra of metal oxides from time-dependent density functional theory and many-body perturbation theory based on optimally-tuned hybrid functionals. Phys. Rev. Mater. 2023, 7 (12), 123803 10.1103/PhysRevMaterials.7.123803.

[ref50] Gant S. E.; Haber J. B.; Filip M. R.; Sagredo F.; Wing D.; Ohad G.; Kronik L.; Neaton J. B. Optimally tuned starting point for single-shot GW calculations of solids. Phys. Rev. Mater. 2022, 6 (5), 053802 10.1103/PhysRevMaterials.6.053802.

[ref51] da Silva Filho D. A.; Coropceanu V.; Fichou D.; Gruhn N. E.; Bill T. G.; Gierschner J.; Cornil J.; BreDas J.-L. Hole-vibronic coupling in oligothiophenes: Impact of backbone torsional flexibility on relaxation energies. Philos. Trans. R. Soc., A 2007, 365 (1855), 1435–1452. 10.1098/rsta.2007.2025. 17428767

[ref52] Körzdörfer T.; Sears J. S.; Sutton C.; Brédas J.-L. Long-range corrected hybrid functionals for π-conjugated systems: Dependence of the range-separation parameter on conjugation length. J. Chem. Phys. 2011, 135, 204107 10.1063/1.3663856. 22128928

[ref53] Bilgiç B.; Kılıç Ç.; Esat B. First-principles study of polyacetylene derivatives bearing nitroxide radicals. Phys. Rev. B 2011, 84 (11), 115207 10.1103/PhysRevB.84.115207.

[ref54] de Queiroz T. B.; Kümmel S. Charge-transfer excitations in low-gap systems under the influence of solvation and conformational disorder: Exploring range-separation tuning. J. Chem. Phys. 2014, 141 (8), 084303 10.1063/1.4892937. 25173010

[ref55] Vlček V.; Eisenberg H. R.; Steinle-Neumann G.; Neuhauser D.; Rabani E.; Baer R. Spontaneous charge carrier localization in extended one-dimensional systems. Phys. Rev. Lett. 2016, 116 (18), 186401 10.1103/PhysRevLett.116.186401. 27203334

[ref56] Su N. Q.; Mahler A.; Yang W. Preserving symmetry and degeneracy in the localized orbital scaling correction approach. J. Phys. Chem. Lett. 2020, 11 (4), 1528–1535. 10.1021/acs.jpclett.9b03888. 32004430 PMC9999725

[ref57] Mei Y.; Yang N.; Yang W. Describing polymer polarizability with localized orbital scaling correction in density functional theory. J. Chem. Phys. 2021, 154, 054302 10.1063/5.0035883. 33557560 PMC8329818

[ref58] Hernangómez-Pérez D.; Gunasekaran S.; Venkataraman L.; Evers F. Solitonics with polyacetylenes. Nano Lett. 2020, 20 (4), 2615–2619. 10.1021/acs.nanolett.0c00136. 32125870

[ref59] Nguyen N. L.; Colonna N.; Ferretti A.; Marzari N. Koopmans-compliant spectral functionals for extended systems. Phys. Rev. X 2018, 8, 021051 10.1103/PhysRevX.8.021051.

[ref60] See Supporting Information at [URL will be inserted by publisher] for more computational details.

[ref61] Savin A.; Flad H.-J. Density functionals for the Yukawa electron-electron interaction. Int. J. Quantum Chem. 1995, 56 (4), 327–332. 10.1002/qua.560560417.

[ref62] Yanai T.; Tew D. P.; Handy N. C. A new hybrid exchange–correlation functional using the Coulomb-attenuating method (CAM-B3LYP). Chem. Phys. Lett. 2004, 393 (1), 51–57. 10.1016/j.cplett.2004.06.011.

[ref63] Perdew J. P.; Ernzerhof M.; Burke K. Rationale for mixing exact exchange with density functional approximations. J. Chem. Phys. 1996, 105 (22), 9982–9985. 10.1063/1.472933.

[ref64] Adamo C.; Barone V. Toward reliable density functional methods without adjustable parameters: The PBE0 model. J. Chem. Phys. 1999, 110 (13), 6158–6170. 10.1063/1.478522.

[ref65] Heyd J.; Scuseria G. E.; Ernzerhof M. Erratum: “Hybrid functionals based on a screened Coulomb potential” [J. Chem. Phys. 118, 8207 (2003)]. J. Chem. Phys. 2006, 124 (21), 219906 10.1063/1.2204597.

[ref66] Refaely-Abramson S.; Sharifzadeh S.; Govind N.; Autschbach J.; Neaton J. B.; Baer R.; Kronik L. Quasiparticle spectra from a nonempirical optimally tuned range-separated hybrid density functional. Phys. Rev. Lett. 2012, 109 (22), 226405 10.1103/PhysRevLett.109.226405. 23368141

[ref67] Cudazzo P.; Tokatly I. V.; Rubio A. Dielectric screening in two-dimensional insulators: Implications for excitonic and impurity states in graphane. Phys. Rev. B 2011, 84 (8), 085406 10.1103/PhysRevB.84.085406.

[ref68] Andersen K.; Latini S.; Thygesen K. S. Dielectric genome of van der waals heterostructures. Nano Lett. 2015, 15 (7), 4616–4621. 10.1021/acs.nanolett.5b01251. 26047386

[ref69] Qiu D. Y.; Da Jornada F. H.; Louie S. G. Screening and many-body effects in two-dimensional crystals: Monolayer MoS_2_. Phys. Rev. B 2016, 93 (23), 235435 10.1103/PhysRevB.93.235435.

[ref70] Henderson T. M.; Janesko B. G.; Scuseria G. E. Generalized gradient approximation model exchange holes for range-separated hybrids. J. Chem. Phys. 2008, 128, 194105 10.1063/1.2921797. 18500854 PMC2812874

[ref71] Iikura H.; Tsuneda T.; Yanai T.; Hirao K. A long-range correction scheme for generalized-gradient-approximation exchange functionals. J. Chem. Phys. 2001, 115 (8), 3540–3544. 10.1063/1.1383587.

[ref72] Perdew J. P.; Burke K.; Ernzerhof M. Generalized gradient approximation made simple. Phys. Rev. Lett. 1996, 77, 3865–3868. 10.1103/PhysRevLett.77.3865. 10062328

[ref73] Potts A.; Price W. C. The photoelectron spectra of methane, silane, germane and stannane. Proc. R. Soc. London, Ser. A 1972, 326 (1565), 165–179. 10.1098/rspa.1972.0003.

[ref74] Baker A.; Baker C.; Brundle C.; Turner D. The electronic structures of methane, ethane, ethylene and formaldehyde studied by high-resolution molecular photoelectron spectroscopy. Int. J. Mass Spectrom. Ion Phys. 1968, 1 (4–5), 285–301. 10.1016/0020-7381(68)85005-3.

[ref75] Linstrom P. J.; Mallard W. G. The NIST chemistry webbook: A chemical data resource on the internet. J. Chem. Eng. Data 2001, 46 (5), 1059–1063. 10.1021/je000236i.

[ref76] Partridge R. H. Vacuum-ultraviolet absorption spectrum of polyethylene. J. Chem. Phys. 1966, 45 (5), 1685–1690. 10.1063/1.1727815.

[ref77] Fujihira M.; Inokuchi H. Photoemission from polyethylene. Chem. Phys. Lett. 1972, 17 (4), 554–556. 10.1016/0009-2614(72)85104-2.

[ref78] Seki K.; Ueno N.; Karlsson U. O.; Engelhardt R.; Koch E.-E. Valence bands of oriented finite linear chain molecular solids as model compounds of polyethylene studied by angle-resolved photoemission. Chem. Phys. 1986, 105 (1–2), 247–265. 10.1016/0301-0104(86)80072-6.

[ref79] Beez M.; Bieri G.; Bock H.; Heilbronner E. The ionization potentials of butadiene, hexatriene, and their methyl derivatives: Evidence for through space interaction between double bond π-orbitals and non-bonded pseudo-π orbitals of methyl groups?. Helv. Chim. Acta 1973, 56 (3), 1028–1046. 10.1002/hlca.19730560321.

[ref80] Jones T.; Maier J. Study of the radical cation of all trans-1, 3, 5, 7-octatetraene by its emission, Ã^2^Au → X̃^2^Bg, and by photoelect. Int. J. Mass Spectrom. Ion Phys. 1979, 31 (3), 287–291. 10.1016/0020-7381(79)83029-6.

[ref81] Frommer J.; Chance R. Encyclopedia of Polymer Science and Engineering; John Wiley & Sons, 1986; Vol. 5, pp 462–507.

[ref82] Jones D.; Guerra M.; Favaretto L.; Modelli A.; Fabrizio M.; Distefano G. Determination of the electronic structure of thiophene oligomers and extrapolation to polythiophene. J. Phys. Chem. A 1990, 94 (15), 5761–5766. 10.1021/j100378a030.

[ref83] Anisimov V. I.; Kozhevnikov A. V. Transition state method and Wannier functions. Phys. Rev. B 2005, 72, 075125 10.1103/PhysRevB.72.075125.

[ref84] Cococcioni M.; de Gironcoli S. Linear response approach to the calculation of the effective interaction parameters in the LDA+U method. Phys. Rev. B 2005, 71, 035105 10.1103/PhysRevB.71.035105.

[ref85] Weng M.; Li S.; Ma J.; Zheng J.; Pan F.; Wang L.-W. Wannier Koopman method calculations of the band gaps of alkali halides. Appl. Phys. Lett. 2017, 111 (5), 054101 10.1063/1.4996743.

[ref86] Bischoff T.; Reshetnyak I.; Pasquarello A. Adjustable potential probes for band-gap predictions of extended systems through nonempirical hybrid functionals. Phys. Rev. B 2019, 99, 201114 10.1103/PhysRevB.99.201114.

[ref87] Bischoff T.; Wiktor J.; Chen W.; Pasquarello A. Nonempirical hybrid functionals for band gaps of inorganic metal-halide perovskites. Phys. Rev. Mater. 2019, 3, 123802 10.1103/PhysRevMaterials.3.123802.

[ref88] Elliott J. D.; Colonna N.; Marsili M.; Marzari N.; Umari P. Koopmans meets Bethe-Salpeter: Excitonic optical spectra without GW. J. Chem. Theory Comput. 2019, 15 (6), 3710–3720. 10.1021/acs.jctc.8b01271. 30998361

[ref89] Weng M.; Pan F.; Wang L.-W. Wannier–Koopmans method calculations for transition metal oxide band gaps. npj. Comput. Mater. 2020, 6 (1), 33 10.1038/s41524-020-0302-0.

[ref90] Bischoff T.; Reshetnyak I.; Pasquarello A. Band gaps of liquid water and hexagonal ice through advanced electronic-structure calculations. Phys. Rev. Res. 2021, 3 (2), 023182 10.1103/PhysRevResearch.3.023182.

[ref91] Colonna N.; De Gennaro R.; Linscott E.; Marzari N. Koopmans spectral functionals in periodic boundary conditions. J. Chem. Theory Comput. 2022, 18 (9), 5435–5448. 10.1021/acs.jctc.2c00161. 35924825

[ref92] Mahler A.; Williams J.; Su N. Q.; Yang W. Localized orbital scaling correction for periodic systems. Phys. Rev. B 2022, 106 (3), 035147 10.1103/PhysRevB.106.035147. 37727592 PMC10508887

[ref93] Yang J.; Falletta S.; Pasquarello A. One-shot approach for enforcing piecewise linearity on hybrid functionals: Application to band gap predictions. J. Phys. Chem. Lett. 2022, 13 (13), 3066–3071. 10.1021/acs.jpclett.2c00414. 35352960

[ref94] De Gennaro R.; Colonna N.; Linscott E.; Marzari N. Bloch’s theorem in orbital-density-dependent functionals: Band structures from Koopmans spectral functionals. Phys. Rev. B 2022, 106 (3), 035106 10.1103/PhysRevB.106.035106.

[ref95] Linscott E. B.; Colonna N.; De Gennaro R.; Nguyen N. L.; Borghi G.; Ferretti A.; Dabo I.; Marzari N. Koopmans: An open-source package for accurately and efficiently predicting spectral properties with Koopmans functionals. J. Chem. Theory Comput. 2023, 19 (20), 7079–7111. 10.1021/acs.jctc.3c00652. PMC10601481 37610300

[ref96] Boys S. F. Construction of some molecular orbitals to be approximately invariant for changes from one molecule to another. Rev. Mod. Phys. 1960, 32 (2), 296 10.1103/RevModPhys.32.296.

[ref97] Foster J. M.; Boys S. Canonical configurational interaction procedure. Rev. Mod. Phys. 1960, 32 (2), 300 10.1103/RevModPhys.32.300.

[ref98] Foster J. M.; Boys S. A quantum variational calculation for hcho. Rev. Mod. Phys. 1960, 32 (2), 303 10.1103/RevModPhys.32.303.

[ref99] Boys S. Localized Orbitals and Localized Adjustment Functions; Academic Press: New York, 1966; pp 253–262.

[ref100] Mostofi A. A.; Yates J. R.; Pizzi G.; Lee Y.-S.; Souza I.; Vanderbilt D.; Marzari N. An updated version of wannier90: A tool for obtaining maximally-localised Wannier functions. Comput. Phys. Commun. 2014, 185 (8), 2309–2310. 10.1016/j.cpc.2014.05.003.

