# Supplementary material for: Nonempirical Prediction of the Length-Dependent Ionization Potential in Molecular Chains
Source: J Chem Theory Comput. 2024 Aug 13;20(16):7168–75. doi: 10.1021/acs.jctc.4c00847 (PMC11360138; doi:10.1021/acs.jctc.4c00847)
Supplement: Supplementary file 1 — ct4c00847_si_001.pdf [file ct4c00847_si_001.pdf]

# Supporting Information: Non-empirical prediction of the length-dependent ionization potential in molecular chains

Guy Ohad,[1] Michal Hartstein,[1] Tim Gould,[2] Jeffrey B. Neaton,[3,4,5] and Leeor Kronik[1]

[1]*Department of Molecular Chemistry and Materials Science, Weizmann Institute of Science, Rehovoth 76100, Israel*
[2]*Queensland Micro- and Nanotechnology Centre, Griffith University, Nathan, QLD 4111, Australia*
[3]*Department of Physics, University of California, Berkeley, Berkeley, California 94720, USA*
[4]*Materials Sciences Division, Lawrence Berkeley National Laboratory, Berkeley, California 94720, USA*
[5]*Kavli Energy NanoSciences Institute at Berkeley, University of California, Berkeley, Berkeley, California 94720, USA*

## S.I. COMPUTATIONAL DETAILS

All calculations were performed using the Vienna *ab initio* simulation package (VASP).[1] We used the PBE-based projector augmented wave (PAW) method for treating core electrons,[2,3] with an energy cutoff of 500 eV throughout. The valence configuration included in the PAWs is $1s^1$ for H, $2s^2 2p^2$ for C and $3s^2 3p^4$ for S. We also made use of the WANNIER90 software package[4] for generating maximally localized Wannier functions, and the AVOGADRO[5] and VESTA[6] softwares for visualization and structural analysis.

### A. Geometry optimization

We optimized the geometry of the molecules using the molecular mechanics tool implemented in AVOGADRO, employing the MMFF94 force field.[7–11] The geometry was not further optimized at the DFT level in order to limit the effect of orbital delocalization to chain length increase alone, similar to Refs.[12,13]. We note that PBE-level optimization leads to lattice distortions that do play a role in orbital localization. These distortions can shift the computed IP by a typical amount of ∼0.1-0.2 eV, but as much as ∼0.4 eV, especially for the longer chains.

### B. Supercells and reciprocal space sampling

Because VASP employs periodic boundary conditions, the chains were studied in tetragonal supercells with large vacuum, where they are oriented in parallel to the $x$-axis. For the finite oligomers we used supercells of $(15 + 5m + f(n)) \times (15+5m) \times (15+5m)$ Å$^3$ for tuning and Wannierization calculations and $(25+f(n)) \times 25 \times 25$ Å$^3$ for calculating the HOMO energy, where $m = 0$ for the alkanes and tOAs and $m = 1$ for the OLTs, and

$$f(n) = \begin{cases} \max[0, 3(n-1)] & \text{Alkanes} \\ 3(n-1) & \text{tOAs} \\ \max[0, 10(n-1)] & \text{OLTs} \end{cases} , \tag{1}$$

and $n$ is the number of repeating units. We used a Γ-point-only sampling of the Brillouin zone.

To calculate the HOMO energy for the infinite polymers, the length along the $x$-axis was set to the length of the repeating units, which are 2.548Å for polyethylene, 2.449 Å for *trans*-polyacetylene, and 7.819 Å for polythiophene. The length along the $y$- and $z$- axis was set to 40 Å for polyethylene and *trans*-polyacetylene and to 50 Å for polythiophene, using a $10 \times 1 \times 1$ $\boldsymbol{k}$-grid sampling. For the Wannierization and tuning calculations of the polymers, the length along the $y$- and $z$- axis was set to 25 Å.

### C. Tuning

The optimally-tuned range-separation parameters obtained based on the OT- and WOT-RSH methods are given in Table S.I. The use of tetragonal unit cells requires the correction of the total energy of the $(N-1)$-electron system with image charge corrections that account for the anisotropy.[14,15] In the WOT calculations we set the Lagrange multiplier to 300 eV for the alkanes and OLTs (including their polymers) and to 400 eV for tOAs (including the polymer), to enforce an occupancy of less then $4 \cdot 10^{-4}$ for the Wannier function.

TABLE S.I: Optimally-tuned range-separation parameters from both OT and WOT, in $\text{Å}^{-1}$.

| | Alkanes | | tOAs | | OLTs | |
|---|---|---|---|---|---|---|
| $n$ | OT | WOT | OT | WOT | OT | WOT |
| 0.5 | 0.72 | 0.75 | - | - | 0.42 | 0.41 |
| 1 | 0.67 | 0.67 | 0.56 | 0.58 | 0.35 | 0.35 |
| 1.5 | 0.60 | 0.64 | - | - | 0.30 | 0.32 |
| 2 | 0.48 | 0.62 | 0.46 | 0.47 | 0.27 | 0.30 |
| 2.5 | 0.45 | 0.62 | - | - | 0.24 | 0.29 |
| 3 | 0.42 | 0.61 | 0.40 | 0.40 | 0.22 | 0.28 |
| 3.5 | 0.41 | 0.61 | - | - | 0.21 | 0.28 |
| 4 | 0.39 | 0.61 | 0.35 | 0.36 | 0.19 | 0.27 |
| 4.5 | 0.38 | 0.60 | - | - | - | - |
| 5 | 0.37 | 0.60 | 0.31 | 0.33 | - | - |
| 6 | 0.36 | 0.60 | 0.28 | 0.30 | - | - |
| 7 | 0.35 | 0.60 | 0.25 | 0.28 | - | - |
| 8 | - | 0.59 | 0.22 | 0.27 | - | - |
| 9 | - | 0.59 | 0.20 | 0.25 | - | - |
| 10 | - | 0.59 | 0.18 | - | - | - |
| $\infty$ | - | 0.49 | - | 0.08 | - | 0.17 |

In the tuning of the polymers, we found that $\Delta I$ converges very slowly with respect to the supercell length along the $x$-axis, denoted $L$. To determine the value of $\Delta I$ in the limit of an infinite chain, we computed $\Delta I$ using a fixed range-separation parameter for an increasingly large $L$. We then fitted $\Delta I$ against $L$ to a function of the form

$$\Delta I(L) = \frac{a}{L^b} + \Delta I(\infty). \tag{2}$$

where $a$ and $b$ are positive constants and $\Delta I(\infty)$ is the asymptotic limit of interest. The tuning was then performed by using a finite $L$ and correcting according to the fit. The fitting curves and parameters are shown in Fig. S.I.

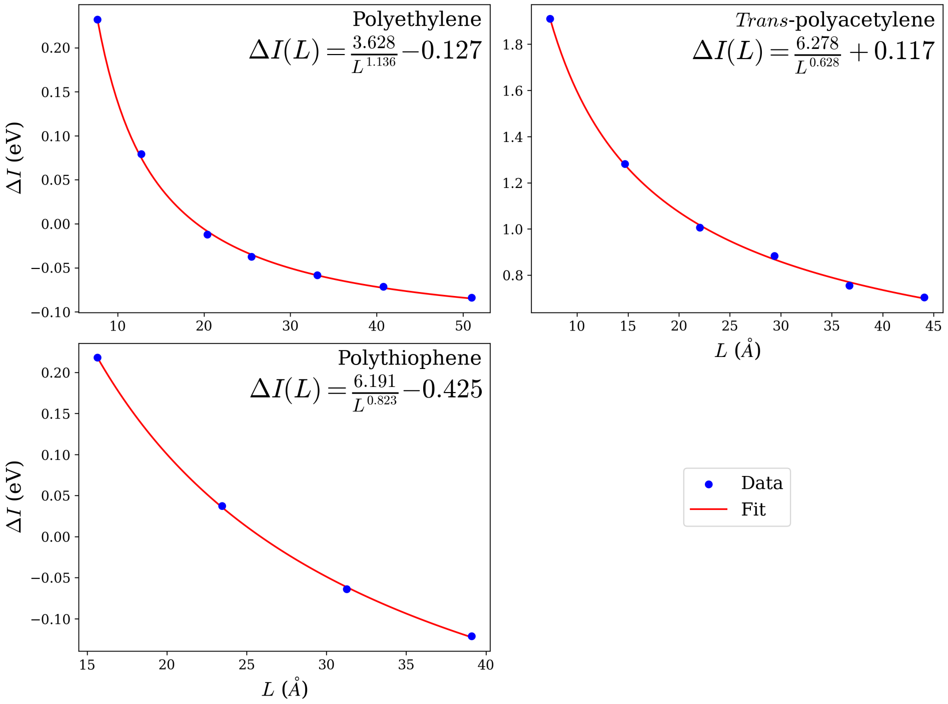


FIG. S.I: Fitting curves and parameters of Eq. (2) for the three polymers.

### D. Orbital spread

We define the spread of an orbital $\varphi$ as[16]

$$R \equiv \left( \langle\varphi| \, r^2 \, |\varphi\rangle - \langle\varphi| \, \hat{\boldsymbol{r}} \, |\varphi\rangle^2 \right)^{1/2}, \tag{3}$$

where $\hat{\boldsymbol{r}}$ is the position operator. For consistency, the spreads for both the HOMOs and Wannier functions are calculated using the WANNIER90 code, and are reported in Table S.II.

TABLE S.II: Spatial spread, in Å, of the HOMO and Wannier function in selected chains.

| | Alkanes | | tOAs | | OLTs | |
|---|---|---|---|---|---|---|
| $n$ | HOMO | Wannier | HOMO | Wannier | HOMO | Wannier |
| 0.5 | 1.1 | 0.8 | - | - | 1.6 | 1.3 |
| 1 | 1.5 | 0.8 | 1.2 | 1.0 | 2.4 | 1.3 |
| 1.5 | 1.6 | 0.8 | - | - | 3.1 | 1.3 |
| 2 | 1.8 | 0.8 | 1.8 | 1.0 | 3.8 | 1.3 |
| 2.5 | 2.0 | 0.8 | - | - | 4.4 | 1.3 |
| 3 | 2.2 | 0.8 | 2.4 | 1.0 | - | 1.3 |
| 3.5 | 2.4 | 0.8 | - | - | 5.7 | 1.3 |
| 4 | 2.6 | 0.8 | 2.8 | 1.0 | 6.3 | 1.3 |
| 4.5 | 2.8 | 0.8 | - | - | - | - |
| 5 | 3.0 | 0.8 | 3.3 | 1.1 | - | - |
| 7 | 3.7 | 0.8 | 4.2 | 1.1 | - | - |
| 8 | 4.1 | 0.8 | 4.6 | 1.1 | - | - |
| 9 | 4.4 | 0.8 | 5.1 | 1.1 | - | - |
| 10 | 4.8 | 0.8 | 5.5 | 1.1 | - | - |

## S.II. COMPARISON BETWEEN HOMO AND WANNIER FUNCTION

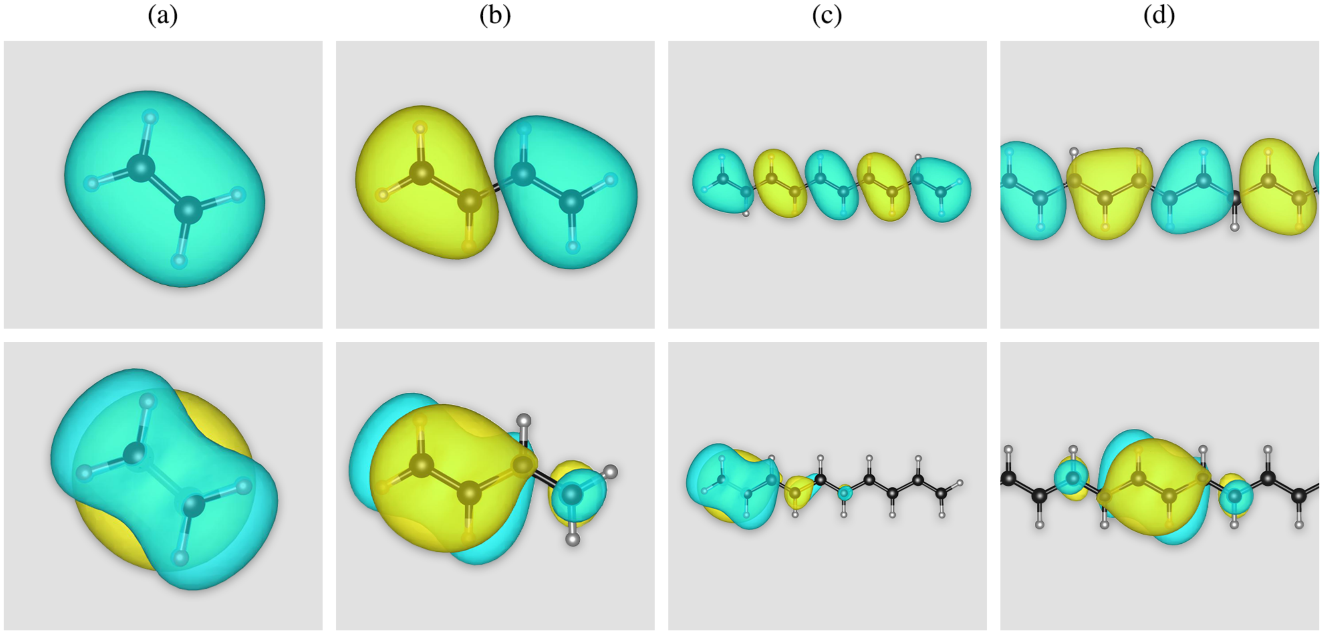


FIG. S.II: HOMO (top row) and highest expectation energy Wannier function (bottom row) for selected tOAs: (a) $n = 1$, (b) $n = 2$, (c) $n = 5$ and (d) $n \to \infty$. Carbon and hydrogen atoms are shown in black and grey, respectively. The wavefunction isosurface is shown in light blue and yellow for a value of 2.0.

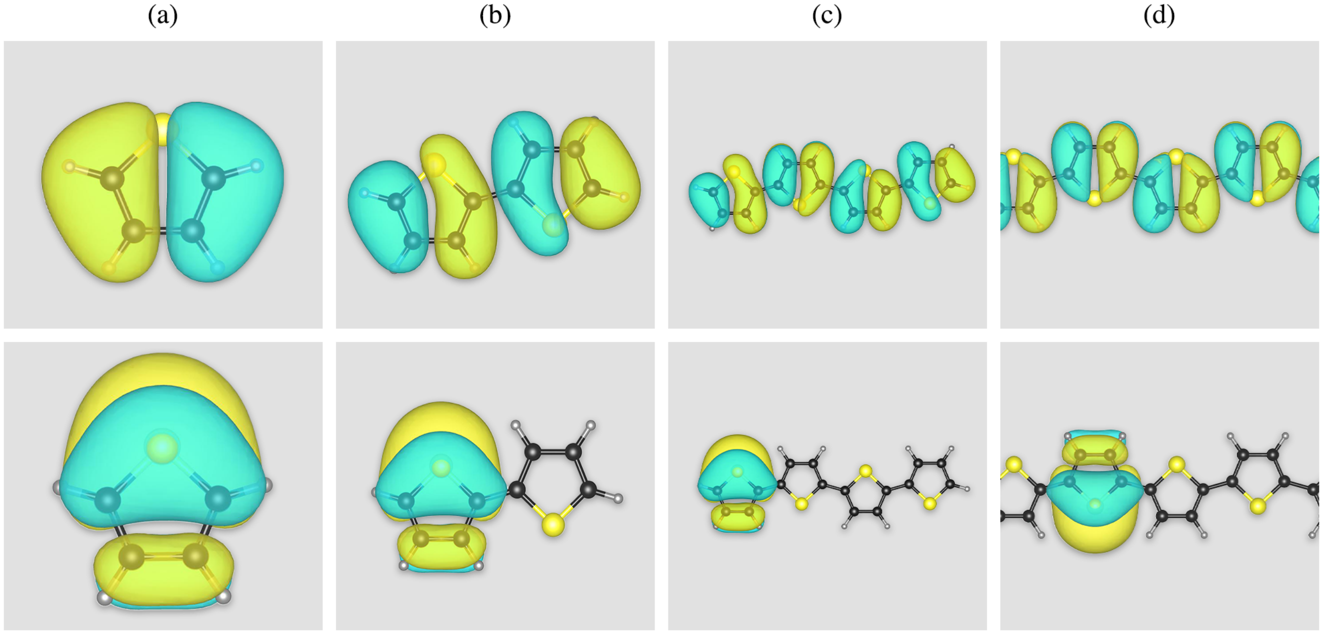


FIG. S.III: HOMO (top row) and highest expectation energy Wannier function (bottom row) for selected OLTs: (a) $n = 0.5$, (b) $n = 1$, (c) $n = 2$ and (d) $n \to \infty$. Carbon, hydrogen, and sulfur atoms are shown in black, grey and yellow, respectively. The wavefunction isosurface is shown in light blue and yellow for a value of 2.6.

[1] Kresse, G. and Furthmüller, J., Efficient iterative schemes for ab initio total-energy calculations using a plane-wave basis set, *Phys. Rev. B* **54**, pp. 11169–11186 (1996).
[2] Blöchl, P. E., Projector augmented-wave method, *Phys. Rev. B* **50**(24), pp. 17953 (1994).
[3] Kresse, G. and Joubert, D., From ultrasoft pseudopotentials to the projector augmented-wave method, *Phys. Rev. B* **59**, pp. 1758–1775 (1999).
[4] Mostofi, A. A., Yates, J. R., Pizzi, G., Lee, Y.-S., Souza, I., Vanderbilt, D., and Marzari, N., An updated version of wannier90: A tool for obtaining maximally-localised Wannier functions, *Comput. Phys. Commun.* **185**(8), pp. 2309 – 2310 (2014).
[5] Hanwell, M. D., Curtis, D. E., Lonie, D. C., Vandermeersch, T., Zurek, E., and Hutchison, G. R., Avogadro: An advanced semantic chemical editor, visualization, and analysis platform, *J. Cheminf.* **4**(1), pp. 1–17 (2012).
[6] Momma, K. and Izumi, F., VESTA 3 for three-dimensional visualization of crystal, volumetric and morphology data, *J. Appl. Crystallogr.* **44**(6), pp. 1272–1276 (2011).
[7] Halgren, T. A., Merck molecular force field. I. Basis, form, scope, parameterization, and performance of MMFF94, *J. Comput. Chem.* **17**(5-6), pp. 490–519 (1996).
[8] Halgren, T. A., Merck molecular force field. II. MMFF94 van der Waals and electrostatic parameters for intermolecular interactions, *J. Comput. Chem.* **17**(5-6), pp. 520–552 (1996).
[9] Halgren, T. A., Merck molecular force field. III. Molecular geometries and vibrational frequencies for MMFF94, *J. Comput. Chem.* **17**(5-6), pp. 553–586 (1996).
[10] Halgren, T. A. and Nachbar, R. B., Merck molecular force field. IV. Conformational energies and geometries for MMFF94, *J. Comput. Chem.* **17**(5-6), pp. 587–615 (1996).
[11] Halgren, T. A., Merck molecular force field. V. Extension of MMFF94 using experimental data, additional computational data, and empirical rules, *J. Comput. Chem.* **17**(5-6), pp. 616–641 (1996).
[12] Vlček, V., Eisenberg, H. R., Steinle-Neumann, G., Neuhauser, D., Rabani, E., and Baer, R., Spontaneous charge carrier localization in extended one-dimensional systems, *Phys. Rev. Lett.* **116**(18), pp. 186401 (2016).
[13] Nguyen, N. L., Colonna, N., Ferretti, A., and Marzari, N., Koopmans-compliant spectral functionals for extended systems, *Phys. Rev. X* **8**, pp. 021051 (2018).
[14] Makov, G. and Payne, M. C., Periodic boundary conditions in ab initio calculations, *Phys. Rev. B* **51**, pp. 4014–4022 (1995).
[15] Rurali, R. and Cartoixá , X., Theory of defects in one-dimensional systems: Application to Al-catalyzed Si nanowires, *Nano Lett.* **9**(3), pp. 975–979 (2009).
[16] Marzari, N., Mostofi, A. A., Yates, J. R., Souza, I., and Vanderbilt, D., Maximally localized Wannier functions: Theory and applications, *Rev. Mod. Phys.* **84**, pp. 1419–1475 (2012).
